# Supplementary figures and images for: Glutamate dehydrogenase from Pantoea ananatis: A new bacterial enzyme with dual coenzyme specificity
Source: PLoS One. 2025 Aug 19;20(8):e0328289. doi: 10.1371/journal.pone.0328289 (PMC12364357; doi:10.1371/journal.pone.0328289)

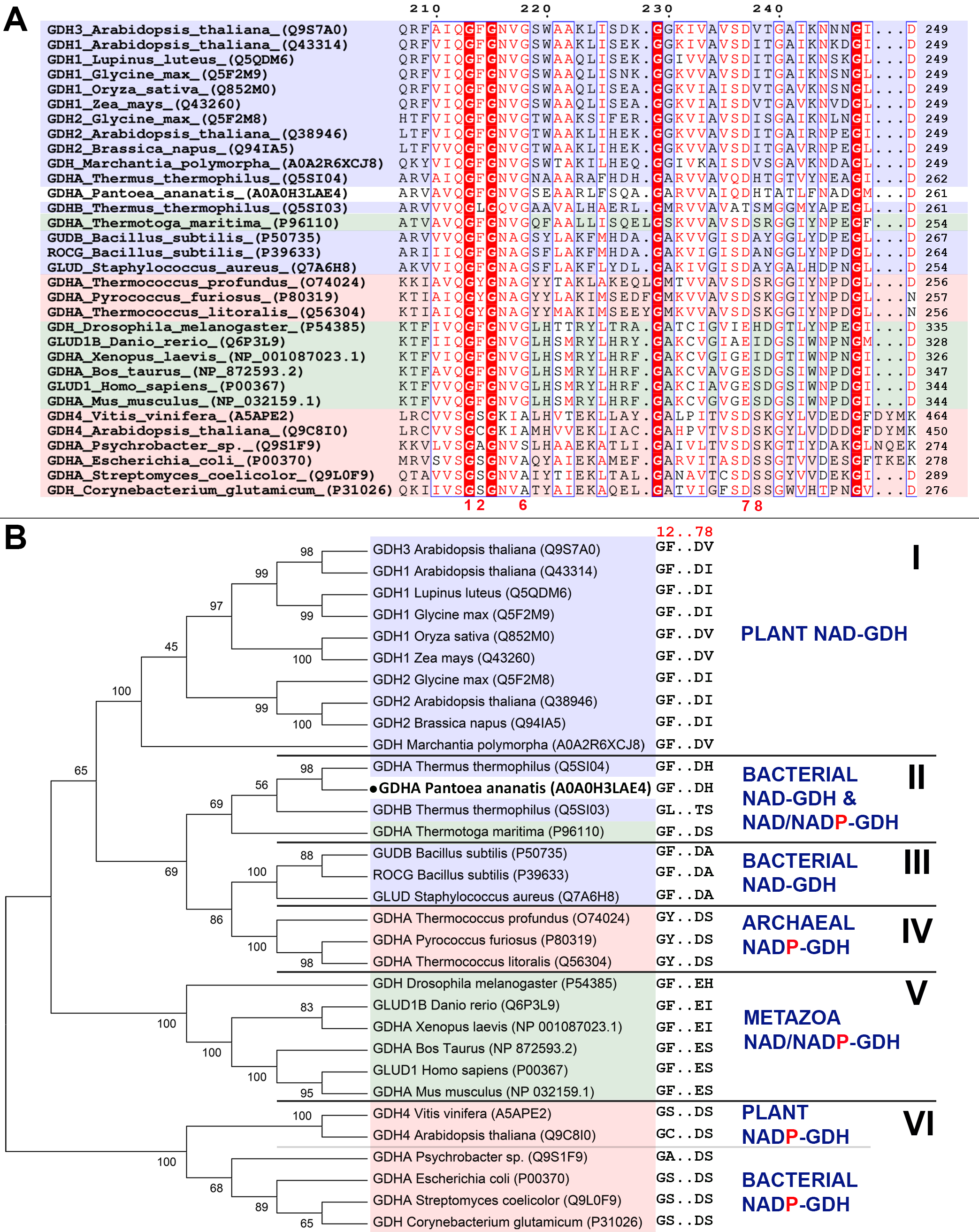

Supplement: S1 Fig — Phylogenetic analysis of GDH protein families. (A) Multiple sequence alignment of conserved coenzyme-binding motif of GdhPa and other species GDHs. The description of each protein sequence includes the protein name and species, and the UniProt or the GenBank accession number (in parentheses). NADP-, NAD- and NAD/NADP-dependent GDHs are highlighted in pink, blue and green, respectively. Completely conserved amino acids are indicated in shaded red boxes. Numbers 1, 2, and 6–8 correspond to the conserved “ﬁngerprint” amino acid residues P1, P2, and P6–P8, respectively. (B) Neighbor-joining tree for 32 GDH50s-group proteins from various species generated by MEGA 12 software. The bootstrap supports (%) calculated from 1,000 replicates are indicated next to the branches. Subfamilies are indicated by Roman numerals (I-VI). (TIF) [file pone.0328289.s001.tif]

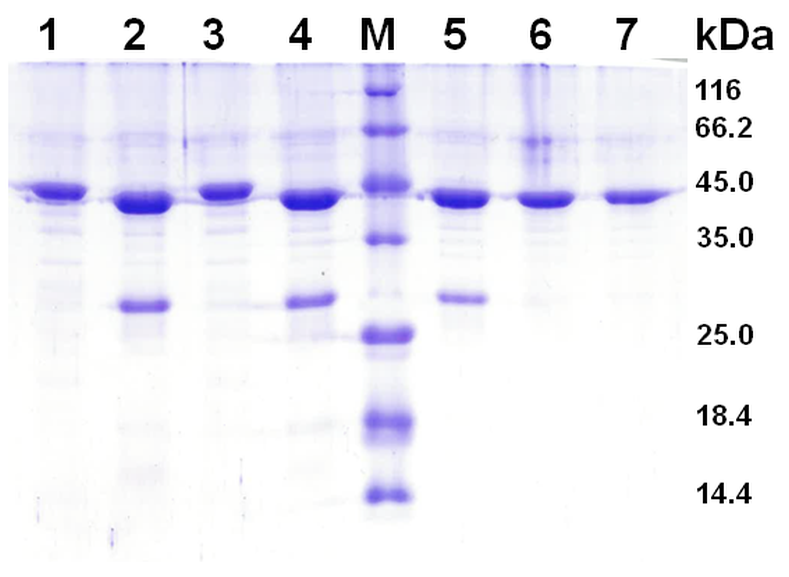

Supplement: S2 Fig — SDS-PAGE analysis of protein fractions of recombinant P. ananatis GDH. SDS-PAGE was performed on a 15% acrylamide gel, and the proteins were visualized using Coomassie Brilliant Blue R-250 staining. Lanes 1, 3: the purified Ht-TEV-GDH_Pan; lanes 2, 4, 5: aliquots of reaction of affinity tag removal from the purified Ht-TEV-GDH_Pan; lanes 6, 7: the purified GdhPa (2 µg and 1.6 µg, respectively); and m: marker proteins. MWs in kDa are shown on the right. (TIF) [file pone.0328289.s002.tif]

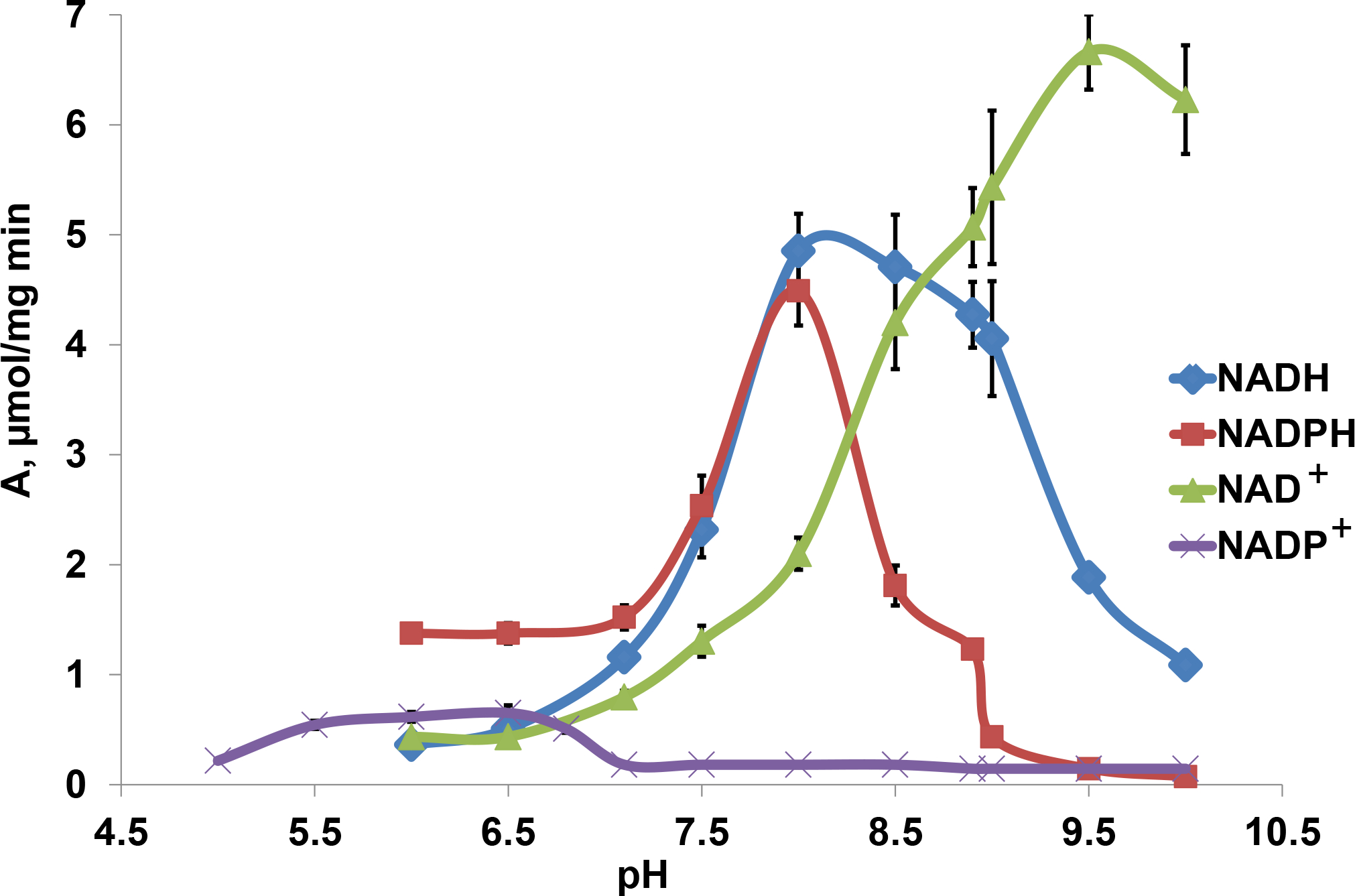

Supplement: S3 Fig — Activity was measured in both NAD(P)H-dependent (reductive amination) and NAD(P)+-dependent (oxidative deamination) reactions in various buffers (100 mM): MES, pH 5.5–6.5; imidazole, pH 6.0–7.5; Tris-HCl, pH 7.1–8.9; and CHES, pH 8.6–10.0. Data are means ± SD of three independent experiments. Some error bars are smaller than the data point symbols. (TIF) [file pone.0328289.s003.tif]

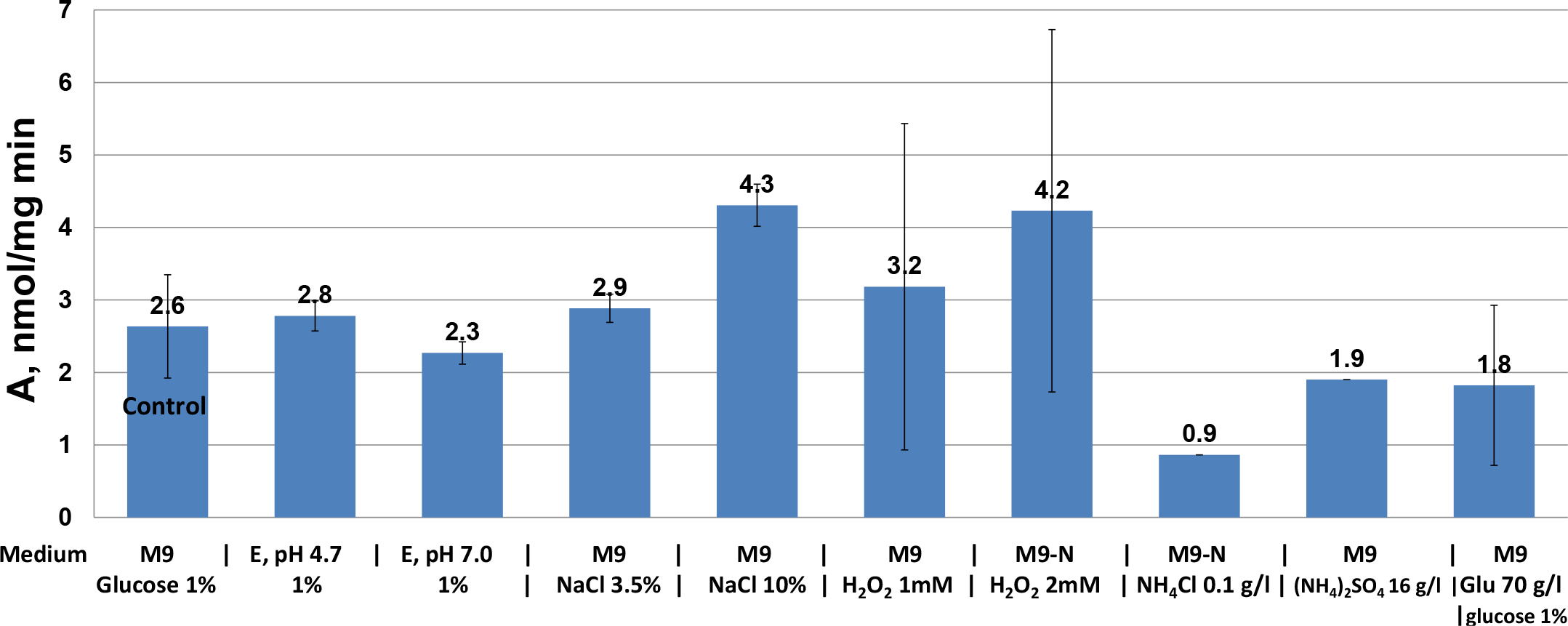

Supplement: S4 Fig — Specific GDH activities (per milligram of total protein) during NADPH-dependent reductive amination of α-KG, measured in cells grown under the conditions indicated (different pH values, high ammonia supply, and addition of inhibitory concentrations of NaCl or H2O2). Data are means ± SD of several independent experiments, n = 3. Brown-Forsythe and Welch ANOVA followed by Dunnett’s post-hoc test were used to determine statistically significant differences between the given group and the control group (Control); p > 0.05, not significant. (TIF) [file pone.0328289.s004.tif]

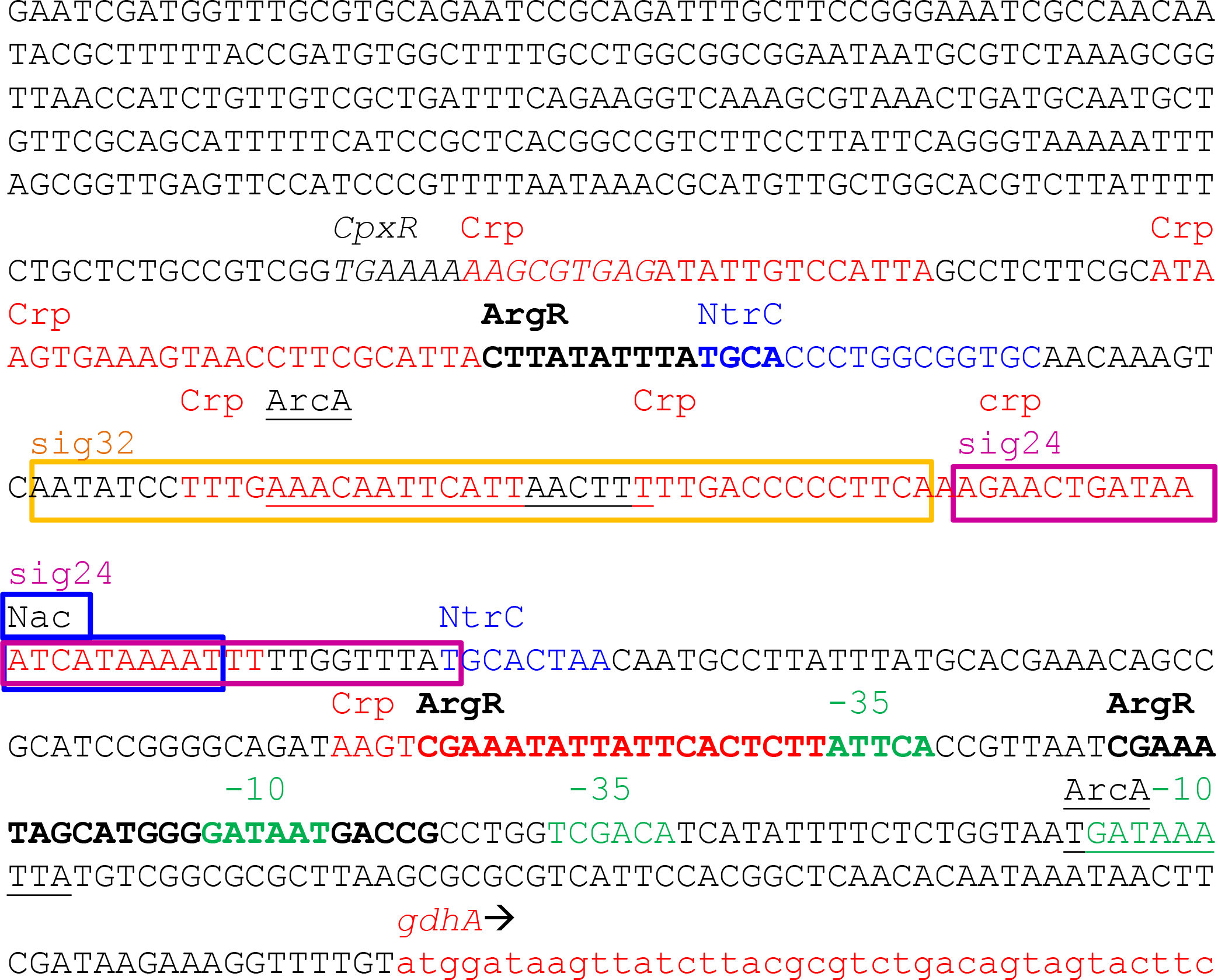

Supplement: S5 Fig — Putative regulatory elements were identified using various in silico tools. Putative −10 and −35 promoter sequence for σ70 (green font), putative promoter sequences σ32 (orange box), and σ24 (pink box) are indicated. Putative regulatory binding sites for CpxR (italics), Crp (red font), ArcA (underlined), ArgR (bold), NtrC (blue font), and Nac (blue boxes) are also marked. The gdhAPa coding sequence is indicated in red lowercase letters. (TIF) [file pone.0328289.s005.tif]
