# Supplementary material for: Glutamate dehydrogenase from Pantoea ananatis: A new bacterial enzyme with dual coenzyme specificity
Source: PLoS One. 2025 Aug 19;20(8):e0328289. doi: 10.1371/journal.pone.0328289 (PMC12364357; doi:10.1371/journal.pone.0328289)
Supplement: S1 Table — (DOCX) [file pone.0328289.s006.docx]

**S1 Table. Primers used in this study.**

| Name | Sequence (5' to 3') | Application |
| --- | --- | --- |
| qRT-gdhA-F | aacgctgacggcatggatatgg | RT-qPCR (*gdhA_Pa_*) |
| qRT-gdhA-R | gccagaaacgctctttctcgatgtt | RT-qPCR (*gdhA_Pa_*) |
| qRT-ffh-R | acgtcgcccatgccaagaatac | RT-qPCR (*ffh*) |
| qRT-ffh-F | atcaccggcaagccgatcaaat | RT-qPCR (*ffh*) |
| qRT-gyrB-R | ttcagcgaccagtgtttccagc | RT-qPCR (*gyrB*) |
| qRT-gyrB-F | atgacgaggccatgctgcag | RT-qPCR (*gyrB* |
| rpsC(2841)-qRCR-F | tatcgagcgtccggctaagagcatc | RT-qPCR (*rpsC*) |
| rpsC(2841)-qRCR-R | tcagcaaccagtttagcgtccagttc | RT-qPCR (*rpsC*) |
| 6His-TEV-R | accctgaaaatacaggttttcggtaccgtgatgatgatgatgatggct | Plasmid pET-15-TEV-GdhA(Pa) |
| gdhA_Pa-TEV-F | ggtaccgaaaacctgtattttcagggtatggataagttatcttacgc | Plasmid pET-15-TEV-GdhA(Pa) |
| gdhAPA-BamHI-R | taggatccttaacccggatagataccgcgatc | Plasmid pET-15-6His-GdhA(Pa) |
| gdhAPA-NdeI-F | aaaacatatggataagttatcttacgcgtctgac | Plasmid pET-15-6His-GdhA(Pa) |
