# Supplementary material for: Glutamate dehydrogenase from Pantoea ananatis: A new bacterial enzyme with dual coenzyme specificity
Source: PLoS One. 2025 Aug 19;20(8):e0328289. doi: 10.1371/journal.pone.0328289 (PMC12364357; doi:10.1371/journal.pone.0328289)
Supplement: S2 Table — (DOCX) [file pone.0328289.s007.docx]

**S2 Table. Comparison of specific GDH activities of purified proteins before (Ht-TEV-GDH-Pan) and after (Gdh_Pa_) affinity tag removal.**

| **Protein** | **Specific activity^1^, U mg^-1^** | | | |
| --- | --- | --- | --- | --- |
|  | **Reductive amination** | | **Oxidative deamination** | |
|  | **NADH-dependent** | **NADPH-dependent** | **NAD^+^-dependent** | **NADP^+^-dependent** |
| **Ht-TEV-GDH-Pan** | 2.25 ± 0.20 | 3.04 ± 0.038 | 2.76 ± 0.51 | 0.34 ± 0.06 |
| **Gdh_Pa_** | 4.71 ± 0.53 | 4.78 ± 0.58 | 7.17 ± 0.88 | 0.58 ± 0.04 |

^1^ Specific GDH activities were determined in at least three independent assays as described in Materials and methods. Statistical analysis was performed using the GraphPad Prism 10 software. Data are mean ± SEM, n≥3.
