# Supplementary material for: Glutamate dehydrogenase from Pantoea ananatis: A new bacterial enzyme with dual coenzyme specificity
Source: PLoS One. 2025 Aug 19;20(8):e0328289. doi: 10.1371/journal.pone.0328289 (PMC12364357; doi:10.1371/journal.pone.0328289)
Supplement: S3 Table — (DOCX) [file pone.0328289.s008.docx]

**S3 Table. Effects of amino acids and purine nucleotides on GDH activity.**

| **Compound** | **Activity (%)^a^** |
| --- | --- |
| None | 100 ± 9 |
| AMP | 98 ± 8 |
| ADP | 96 ± 8 |
| ATP | 90 ± 9 |
| GMP | 100 ± 3 |
| GDP | 100 ± 6 |
| GTP | 106 ± 8 |
| IMP | 108 ± 9 |
| L-proline | 111 ± 8 |
| L-arginine | 103 ± 5 |
| L-leucine | 121 ± 12 |
| L-leucine* | 183 ± 15 |
| L-valine | 114 ± 10 |
| L-phenylalanine | 104 ± 6 |

^a^ 100% denotes 7.17 U mg^-1^ activity of Gdh_Pa_ in NAD^+^-dependent oxidative deamination without effectors. The reaction mixture (0.5 ml) contained 80 mM buffer (CHES, pH 9.5, 150 mM Glu (except for the line marked with an asterisk, where the Glu concentration was 10 mM), 2.0 mM NAD^+^ and purified recombinant Gdh_Pa_ (1.11 µg). Possible effectors (AMP, ADP, ATP, GMP, GDP, GTP or IMP, 1 mM each; or amino acids L-proline, L-arginine, L-leucine, L-valine or L-phenylalanine, 10 mM each) were added in reaction mixture. Specific GDH activities were determined in at least three independent assays. Data are mean ± SEM, n=3.
