# Supplementary material for: Glutamate dehydrogenase from Pantoea ananatis: A new bacterial enzyme with dual coenzyme specificity
Source: PLoS One. 2025 Aug 19;20(8):e0328289. doi: 10.1371/journal.pone.0328289 (PMC12364357; doi:10.1371/journal.pone.0328289)
Supplement: Attachment — Submitted filename: Response_to_Reviewers_auresp_2.docx [file pone.0328289.s011.docx]

We appreciate the time and effort invested by the academic editor and reviewers in evaluating our manuscript.

Journal Requirements:

Please review your reference list to ensure that it is complete and correct. If you have cited papers that have been retracted, please include the rationale for doing so in the manuscript text, or remove these references and replace them with relevant current references. Any changes to the reference list should be mentioned in the rebuttal letter that accompanies your revised manuscript. If you need to cite a retracted article, indicate the article’s retracted status in the References list and also include a citation and full reference for the retraction notice.

**Answer.** We have carefully reviewed the reference list and in-text citations to ensure compliance with the journal’s guidelines. After some corrections in the text and thorough verification, we confirm that the reference list does not contain any retracted articles.

In response to the reviewer’ comments, the following additional corrections have been made:

1. Additional References: We have incorporated several new references to strengthen the manuscript.

2. Updated Numbering: Due to these additions, the in-text citation numbers and the reference list order have been adjusted accordingly.

All modifications, including changes to citations and the reference list, are clearly highlighted in the tracked-changes version of the manuscript ("Revised Manuscript with Track Changes").

Reviewers' comments:

Reviewer #1: (No Response)

Reviewer #2: Overall, the Authors responded to the comments, and the manuscript has improved significantly as a result.
However, they missed to address one of my major concerns.
In fact, given the complexity of the functional role of GDH enzyme, which is still to be clarified, it is necessary that the authors provide at least a glimpse into its evolutionary features. That's why I suggested to improve the literature by mentioning thre of the most relevant and complete publications (Kwinta, J., Bielawski, W. Glutamate dehydrogenase in higher plants. Acta Physiol Plant 20, 453–463 (1998). https://doi.org/10.1007/s11738-998-0033-1. Tercé-Laforgue et al. Resolving the role of plant glutamate dehydrogenase: II. Physiological characterization of plants overexpressing the two enzyme subunits individually or simultaneously. Plant Cell Physiol. 2013 Oct;54(10):1635-47. doi: 10.1093/pcp/pct108. Brambilla et al. Glutamate dehydrogenase in “Liverworld”—A study in selected species to explore a key enzyme of plant primary metabolism in Marchantiophyta. Physiologia Plantarum 2023, 175, doi: 10.1111/ppl.14071). Despite that, Authors only add one of the suggestions, completely ignoring the sense of the comment itself. Thus, the introduction and the discussion about the conserved aminoacids in functionally critical positions are not adequately informative.

**Answer.** We thank Reviewer #2 for their careful reading of our manuscript and for valuable recommendations to improve it. We apologize for interpreting the initial comment (“The Introduction, as well as the literature, require improvement: mention of plant GDHs and their evolution must be included”) too literally.

We have now addressed the reviewer’s request by the following:

1. Citing all three recommended articles.

2. Expanding the discussion on the evolution of GDH enzymes in different species (lines 55–60, 67–86; 496–498).

3. Adding a new S1 Fig, which presents a phylogenetic tree of glutamate dehydrogenases from various organisms, including plants, animals, bacteria, and Archaea. We also discuss the phylogenetic clustering of *P. ananatis* GDHs within a NAD/NADP-dependent clade distinct from NADP-specific GDHs, possibly indicating horizontal gene transfer (lines 344–359, 902–911).

We believe the revised manuscript now addresses the reviewers' concerns while maintaining clarity and scientific rigor. Please let us know if further modifications are required.

Thank you for your time and consideration.
